# Antimicrobial activity of some sulfonamide derivatives on clinical isolates of Staphylococus aureus

**DOI:** 10.1186/1476-0711-7-17

**Published:** 2008-08-20

**Authors:** Yeliz Genç, Reşit Özkanca, Yunus Bekdemir

**Affiliations:** 1 Department of Biology, Faculty of Science and Arts, Ondokuz Mayis University, Kurupelit, 55139, Samsun, Turkey; 2 Department of Chemistry, Faculty of Science and Arts, Ondokuz Mayis University, Kurupelit, 55139, Samsun, Turkey

## Abstract

**Background:**

*Staphylococcus aureus *is a non-motile, gram positive, non-sporforming, facultative anaerobic microorganism. It is one of the important bacteria as a potential pathogen specifically for nosocomial infections. The sulfonamide derivative medicines are preferred to cure infection caused by *S. aureus *due to methicillin resistance.

**Methods:**

Antimicrobial activity of four sulfonamide derivatives have been investigated against 50 clinical isolates of *S. aureus *and tested by using MIC and disc diffusion methods. 50 clinical isolate which collected from specimens of patients who are given medical treatment in Ondokuz Mayis University Medical School Hospital. A control strain of *S. aureus *ATCC 29213 was also tested.

**Results:**

The strongest inhibition was observed in the cases of **I **[*N*-(2-hydroxy-4-nitro-phenyl)-4-methyl-benzensulfonamid], and **II **[*N*-(2-hydroxy-5-nitro-phenyl)-4-methyl-benzensulfonamid] against *S. aureus*. Compound **I **[*N*-(2-hydroxy-4-nitro-phenyl)-4-methyl-benzensulfonamid] showed higher effect on 21 *S. aureus *MRSAisolates than oxacillin antibiotic. Introducing an electron withdrawing on the ring increased the antimicrobial activity remarkably.

**Conclusion:**

This study may help to suggest an alternative possible leading compound for development of new antimicrobial agents against MRSA and MSSA resistant *S. aureus*. It was also shown here that that clinical isolates of 50 *S. aureus *have various resistance patterns against to four sulfonamide derivatives. It may also be emphasized here that in vitro antimicrobial susceptibility testing results for *S. aureus *need standardization with further studies and it should also have a correlation with in vivo therapeutic response experiments.

## Background

*Staphylococcus aureus *is one of the most significant human pathogens responsible for nosocomial and community acquired infections. It can cause a range of infectious disease from mild conditions, such as soft tissue infections, to severe life-threatening debiliation, such as endocarditis [1]. Despite the recent staphylococci infections, they are persisting as an important hospital and community pathogen [2]. Methicillin resistance has become a major concern to the medical community due to the fact that they have an extraordinary ability to adapt rapidly to antibiotic stress [3]. Among hospital isolates the frequency of methicillin resistant *S. aureus *(MRSA) is very high [4]. There is need to have new chemicals for treatment of staphylococci infections.

The sulfonamides have, for many years, being widely studied for their chemotherapeutic activity. Their important role as antibacterial, antimalarial and antileprotic agents is well recognized [5,6]. Recently, certain sulfonamides have been reported as showing interesting the antibacterial properties of sulfonamides have been extensively studied by Quantitive Structure-activity Relationship&Molecular Modeling (QSAR) method. [7]. Antimicrobial therapy for infections with *S. aureus *often includes sulfonamides which are use to cure nosocomial infections [1]. Sulfonamides are still an alternative option in order to cure methicillin resistant *S. aureus *(MRSA) staphylococci infections. Although the sulfonamide therapy has been reduced, owing to development of more effective antimicrobial agents and to the gradual increase in the resistance of bacterial species, clinical treatment with sulfonamides has undergone a revival by the combination sulfomethoxazole and trimethoprim.

Considering this background, the objective of this study some sulfonamides derivatives were tested in terms of antimicrobial activity with the purpose of revealing possible leading compounds for development of new antimicrobial agents against methicillin resistant *S. aureus *(MRSA) and methicillin sensitive *S. aureus *MSSA.

## Methods

### Preparation of the sulfonamides

General procedure for preparation of the sulfonamides is as follows [8]. For a typical run; 0.06 mol substituted aniline was dissolved in 30 ml benzene. 0.06 mol *p*-toluenesulfonylchloride in 20 ml benzene was added into the solution. 0.06 mol dry pyridine was added into 20 ml benzene slowly and it was refluxed for 4 h, so the solvent was removed and a solid was obtained. The solid was dissolved in 10% (w/II) NaOH solution and extracted with CHCl_3_. Aqueous solution was acidified with HCl to obtain raw sulfonamide. Recrystallization of ethanol-water mixture from raw sulfonamide resulted in corresponding sulfonamide in pure form [9]. Some physical and spectral data of the synthesized sulfonamides were summarized below:

***N- *(2-*Hydroxy*-4-*nitro-phenyl*)-4-*methyl-benzenesulfonamide *(I) **m.p. 181–182°C. ^1^H NMR (acetone-*d*_6_), *δ*(ppm) 2.23 (s,3H), 3.37 (s,1H), 7.34 (d,2H), 7.63 (d,2H), 7.71 (dd,1H), 7.82 (d,2H), 8.65 (s,1H), 10.99 (s,1H); IR (KBr) 3608 (OH), 3270 (NH), 3079 (Ar-H), 2920, 1596, 1525 (NO_2 _asym.), 1446, 1402, (SO_2 _asym.), 1336 (NO_2 _sym.), 1270, 1162, 1128 (SO_2 _sym.) cm^-1 ^[3].

***N*-(2-*Hydroxy*-5-*nitro-phenyl*)-4-*methyl-benzenesulfonamide *(II) **m.p. 208–209°C. ^1^H NMR (acetone-*d*_6_), *δ *(ppm) 2.32 (s,3H), 3.60 (broad,1H,-NH), 6.97 (d,1H), 7.31 (d,2H), 7.75 (d,2H), 7.87 (dd,1H), 8.33 (d,1H), 8.55 (broad,1H,-OH); IR (KBr) 3407 (OH), 3280 (NH), 3085 (Ar-H), 2930, 1596, 1523 (NO_2 _asym.), 1454, (SO_2 _asym.), 1342 (NO_2 _sym.), 1164 (SO_2 _sym.) cm^-1 ^[4].

***N*-(5-*Chloro*-2-*hydroxy-phenyl*)-4-*methyl-benzenesulfonamide *(III) **m.p. 189–190°C. ^1^H NMR (acetone-*d*_6_), *δ *(ppm) 2.35 (s,3H), 3.55 (broad,1H,-NH), 6.79 (d,1H), 6.92 (dd,1H), 7.31 (d,2H), 7.36 (d,1H), 7.71 (d,2H), 8.62 (broad,1H,-OH); IR (KBr) 3450 (OH), 3259 (NH), 3080, 2930, 1602, 1504, 1440, 1384, 1319 (SO_2 _asym.), 1216, 1170 (SO_2 _sym.) cm^-1 ^[3].

***N*-(2-*Hydroxy*-5-*methyl-phenyl*)-4-*methyl-benzenesulfonamide *(IV)) **m.p. 142–143°C. ^1^H NMR (acetone-d_6_), *δ *(ppm) 2.14 (s,3H), 2.33 (s,3H), 3.47 (broad,1H,-NH), 6.67 (d,1H), 6.74 (dd,1H), 7.13 (s,1H), 7.27 (d,2H), 7.67 (d,2H), 8.46 (broad,1H,-OH); IR (KBr) 3370 (OH), 3247 (NH), 3039, 2917, 1596, 1517, 1446, 1390, 1321 (SO_2 _asym.), 1290, 1245, 1187, 1162, 1112 (SO_2 _sym.) cm^-1 ^[3].

### Bacterial strains and inoculums preparation

As a preliminary screening for antimicrobial activity of 4 sulfonamides were tested against 30 meticillin resistant (MRSA) and 20 susceptible (MSSA) clinical isolates of *Staphylococcus aureus *provided by Ondokuz Mayis University, Medical School Department of Microbiology and Infectious disease. MRSA isolates were determined by oxacillin test. The commonly used method in routine laboratory practice for the detection of methicillin resistance is oxacillin disc diffusion. All the clinical isolates were isolated from patients in the hospital. Each *Staphylococcus aureus *isolates were cultured in nutrient broth before the antimicrobial activity test performed. Each isolate was checked for its purity and several colonies were emulsified into 50 ml nutrient broth (LabM). The inoculated flasks were incubated at 37C for 18 h on a rotary shaker at 150 rpm (GFL 3032). Reference strain of *Staphylococcus aureus *ATCC 29213 was used as control strain reference strain in order to monitor the antimicrobial disc susceptibility test [10].

### Antimicrobial activity Screening

Antimicrobial studies were performed according to agar disc diffusion method [11]. To obtain more significant information as to the antibacterial potency of sulfonamides derivatives compound I, II, III, and IV against *Staphylococcus aureus*, subcultures were carried out and minimal bactericidal concentration were determined. The following test conditions were applied; all the compounds were dissolved in dimethylsulfoxid (DMSO, Merck). Sensitest agar (Oxoid) plates were prepared and dried at 35–36°C for about 30 min in an incubator. Test strains were spreaded on solid sensitest agar surface by using sterile swap. Spreaded inoculums were 3.5 × 10^5 ^colony forming unit/ml^-1 ^(0.5 McFarland standards, Biomeriux Colorimeter). At the same time, absorbent paper discs were placed on agar surface (5 mm for compounds and 6 mm for antibiotics)and impregnated with known concentrations which determined previously by MIC tests (500 μg for each disc). Oxacillin 1 μg (Oxoid) and Trimetoprim-Sulfametaksazol 23.75 μg were also used for all test microorganisms as positive control. Blank test showed that DMSO in the preparations of the test solutions does not affect the test organisms. They were inverted and allowed to incubate at 37°C. The inhibition zone around the disc was calculated edge to edge zone of confluent growth which is usually corresponds to the sharpest edge of the zone and to be measured diameter in millimeter. All tests were repeated tree times and average data taken as final result.

## Results and discussion

Methicillin-resistant staphylococci are resistant to all other penicillin, carbapenems, cephems and beta-lactam, beta-lactam inhibitor combinations [11]. Consequently these antibiotics should not be used for treating of methicillin-resistant staphylococci infections. Moreover, recently several studies have shown that the methicillin-resistant staphylococci have started to gain resistance to some other widely used antibiotics (quinolone, macrolide group antibiotics, amino glycosides, tetracycline, trimetoprim sulphamethoxazole (SXT), clindamicin, chloramphenicol as well [12-15]. The resistance increase trimetoprim-sulphametoxazole, which is an alternative medicine in the treatment of methicillin-resistant staphylococci infections, is recently received attention. Previously, trimetoprim-sulphametoxazole resistance has been shown to be 10–53% in Turkey while it was reportedly higher (47–79%) in European countries [16]. Trimetoprim-sulphamethoxazole is used for the treatment of staphylococci infections. SXT has still maintain its' an alternative antibiotic potential for the treatment of MRSA and MSSA infections [1]. In addition, sulphametizol, sulphamethaxozole or sulphisoxazole have been using for the treatment of *E. coli *urinary infection as a single antibiotics [1].

In this study, the in vitro antibacterial activity properties of the compounds tested on clinical isolates of 30 MRSA and 20 MSSA *S. aureus *by using new sulfonamide derivative compounds namely *N*-(2-Hydroxy-4-nitro-phenyl)-4-methyl-benzenesulfonamide(I), N-(2-Hydroxy-5-nitro-phenyl)-4-methyl-benzenesulfonamide, N-(5-Chloro-2-hydroxy-phenyl)-4-methyl-benzenesulfonamide(III>), N-(2-Hydroxy-5-methyl-phenyl)-4-methyl-benzenesulfonamide(IV). Some of the sulfonamides were found to be effective on *S. aureus *among the others. The strongest inhibition was detected by the effect of (N-(2-Hydroxy-4-nitro-phenyl)-4-metil-benzensulfonamide) (**I**). The similar results were obtained in previous studies against *Nocardia *species by the treatment of the same sulfonamide compound (Isik and Özdemir-Koçak, article in press in Microb. Res.)

First of all, we tried to find out (Minimal Inhibitory Concentrations) MIC values of sulfonamides derivatives against *S. aureus *isolates. The rate of MIC values showed alterations from 32 to 512 μg for 50 isolates. All data are given in Table 1 indicated that MIC values was not the same for all isolates i.e. showed variations in terms of resistance. It was given in the literature that treatment of some infections 300 μg sulfafurazol and sulfisoxazole ST are applied to patients [10]. After determining the minimum and maximum MIC values, suitable concentration was selected for all isolates in order to proper comparison. It means, MIC values given in table 1 showed that sulfonamide derivatives I and II given here area potential antibacterial substances. It was also reported that I, and II numbered substances showed strong antibacterial activity against *Staphylococcus aureus *ATTCC 43300 and some other gram positive bacteria [17]. Here tested compounds I, II, and III showed antibacterial activity against reference strain *S. aureus *ATTCC 29213 and their concentration were 32, 64 and 128 μg respectively. Compound I was found as a strongest antibacterial agent against *S. aureus *according to MIC values. The MIC values showed some alterations according to *S. aureus *strains given in Table 1.

**Table 1:** MIC values of sulfonamides derivatives I, II, and III against total 50 *S. aureus *clinical isolates (data given as percentage).

**Compound I**		**Compound II**	**Compound III**
MICs (μg)	Number of susceptible Isolates (%)	Number of susceptible Isolates (%)	Number of susceptible Isolates (%)

32	3 (6)	0 (0)	0 (0)
**64**	14(28)	6 (12)	0 (0)
**128**	19 (38)	39 (78)	18 (36)
**256**	12 (24)	5 (10)	22 (44)
**512**	2 (4)	0 (0)	10 (20)

Compund IV showed very low activity therefore it was not taken in consideration.

Secondly, antimicrobial activity of compounds was tested according to disc diffusion method on the base of MIC values. 500 μg concentrations were chosen as a suitable concentration which showed an effect to all *S. aureus *isolates. All data related to inhibition zones against *S. aureus *were given in Table 2. As regard to results the strongest inhibition was observed in case of compound I. In general, the ratio of inhibition caused by Compound I, II and III were 84%, 50% and 36% respectively (Table 3).

**Table 2:** Measured inhibition zone in diameter (mm) of sulfonamide derivatives (I-IV) (500 μg) against clinical isolates of *Staphylococcus aureus*.

**S. aureus Isolation Number**	**Compound II**	**Compound II**	**Compound III**	**Compound IV**	**OX (mm)**	**SXT (mm)**
**SAY01(MRSA)**	20	14	5	0	0	24
**SAY02(MRSA)**	22	15	11	0	0	25,5
**SAY03(MRSA)**	26	17	10	0	0	28,5
**SAY04(MSSA)**	19	17	13	0	13,5	28,5
**SAY05(MSSA)**	23	18	16	0	13,5	28
**SAY06(MRSA)**	23	18	10	8	0	26
**SAY07(MRSA)**	25	18	19	8	0	25
**SAY08(MRSA)**	26	19	13	0	0	26
**SAY09(MRSA)**	19	16	10	0	0	24
**SAY10(MRSA)**	25	17	11	7	0	23
**SAY11(MRSA)**	25	13	11	9	0	27
**SAY12(MRSA)**	29	19	11	9	0	25,5
**SAY13(MRSA)**	13	14	10	9	0	25
**SAY14(MRSA)**	23	17	15	8	0	25
**SAY15(MRSA)**	21	14	14	0	0	26,5
**SAY16(MRSA)**	25	18	13	0	0	27
**SAY17(MSSA)**	21	18	15	0	21	28,5
**SAY18(MRSA)**	25	16	13	0	0	23,5
**SAY19(MSSA)**	21	16	16	0	18,5	27
**SAY20(MRSA)**	26	15	15	0	0	25
**SAY21(MSSA)**	19	14	13	0	17,5	30,5
**SAY22(MRSA)**	23	14	15	0	0	27
**SAY23(MSSA)**	20	16	13	9	20,5	28
**SAY24(MSSA)**	14	15	14	0	17,5	30
**SAY25(MSSA)**	12	20	10	0	26	31
***S. aureus ***ATCC 29213	26	17	12	10	20	31
**SAY26(MSSA)**	28	25	15	0	23	32,5
**SAY27(MSSA)**	19	11	14	9	18	28
**SAY28(MRSA)**	28	19	11	8	0	26,5
**SAY29(MRSA)**	18	17	10	10	0	26,5
**SAY30(MRSA)**	20	13	12	0	0	23,5
**SAY31(MRSA)**	26	20	14	0	0	26
**SAY32(MSSA)**	21	15	14	10	19	26,5
**SAY33(MSSA)**	18	12	14	11	19	28
**SAY34(MRSA)**	16	18	10	10	0	23,5
**SAY35(MSSA)**	20	22	14	0	17	30
**SAY36(MSSA)**	25	16	16	9	20,5	30
**SAY37(MRSA)**	13	15	11	8	0	22
**SAY38(MRSA)**	27	17	10	0	0	25
**SAY39(MRSA)**	15	14	10	0	0	25,5
**SAY40(MSSA)**	18	16	11	0	18	25,5
**SAY41(MSSA)**	27	19	10	11	13	25,5
**SAY42(MSSA)**	18	17	14	8	22,5	30
**SAY43(MRSA)**	21	11	14	9	0	28
**SAY44(MRSA)**	21	16	11	0	0	27,5
**SAY45(MSSA)**	17	18	11	0	18,5	30
**SAY46(MSSA)**	14	11	12	0	14,5	21,5
**SAY47(MRSA)**	22	13	12	8	0	26,5
**SAY48(MRSA)**	14	18	11	0	0	25,5
**SAY49(MRSA)**	18	17	12	0	0	27
**SAY50(MSSA)**	21	22	12	0	20	31
***S. aureus ***ATCC 29213	26	17	12	10	20	31

MRSA (methicillin-resistance *S. aureus*), MSSA (Methicillin Susceptible *S. aureus*). OX (Oxacillin), SXT (trimetoprim-sulphamethoxazole)

**Table 3:** Susceptibility percentage and median zone diameter (mm) of *Staphylococcus aureus *isolates against sulfonamide derivatives and some antimicrobials determined by disc diffusion method.

Antimicrobials		Inhibition zone diameter (mm)^a^			Zone diameter (mm)		Tested *S. aureus *clinical isolates		
Susceptibility range (%) (n = 50)^b^									

Name	Concentration (μg)	R	I	S	Range	Median	R	I	S^c^

Oxacillin	1 μg	≤10	11–12	≥13	0–23	11.5	30(60)	0	20(40)
Trimetoprim (SXT)	25 μg	≤10	11–15	≥16	21.5–32.5	27	0	0	50(100)
Compound I	500 μg	≤12	13–16	≥17	12–28	20	1(2)	7(14)	42(84)
Compound II	500 μg	≤12	13–16	≥17	11–25	18	3(6)	22(44)	25(50)
Compound III	500 μg	≤12	13–16	≥17	11–19	15	13(26)	19(38)	18(36)
Compound IV	500 μg	≤12	13–16	≥17	0–11	5.5	50(100)	0	0

^a^Zone of inhibition diameter (mm) by disc diffusion method susceptibility testing interpretavive guidelines based on NCCLS 2003.

^b^Number of parentheses indicate the total number of clinical *S. aureus *isolates tested.

^c^S = susceptible; I = intermediate; R = resistant

^d^Trimetoprim-sulphamethoxazole (SXT) is being taken as a positive control for approximate comparision compounds data

Early and recent researchers have suggested that sulfonamides are useful for the treatment of some staphylococci infections, especially against urinary infections [1]. In this study, some sulfonamides derivatives were tested in terms of antimicrobial activity with the purpose of revealing possible leading compounds for the development of new antimicrobial agents. Outcome of the study showed that sulfonamide derivatives I and II have proved to be effective enough, which is comparable with previous studies [17]. It was reported that, sulfonamide **I **and **II **showed the highest inhibitory effect on gram positive bacteria i.e. *S. aureus, N. asteroides, N. farcinia and B. subtilis*. On the contrary, they did not lead to significant inhibitory effect on gram negative bacteria and also yeast and mould namely *E. coli, P. aeruginosa, E. cloaceae*, a yeast *C. albicans *and a mould *A. niger *[17].

Many attempts have been made to relate the antibacterial behavior of sulfonamides with molecular structure [6]. It was shown here that antimicrobial activities of the sulfonamides were increased when introducing electron withdrawing groups into the benzene ring of the compounds (Table 2). The compound I has NO_2 _group in para position according to the sulfonamide group which showed the strongest effect on the *S. aureus*. Ionization is important factor on antimicrobial effect of sulfonamides due to increasing solubility. The behaviors of o-substituted acids are often anomalous [18]. Their strength is sometimes found to be much greater than expected due to direct interaction between the adjacent groups. For example, *o*-hydroxybenzoic acid is 10 times stronger than *p*-hydroxybenzoic acid. In the case of the sulfonamides, possibly the OH group in the *ortho *position stabilize the developing negative charge on the nitrogen through intramolecular hydrogen bonding, and in this way the ionization increases. As can be seen in table 2 the compound I is more effective against the bacteria possibly due to increasing ionization of N-H group which is enhanced by p-NO_2 _group. OH group in ortho position is also supports ionization by involving intramolecular hydrogen bonding.

Introducing NO_2 _group to meta position (compound II) reduces antimicrobial activity in general this is because of lesser effect of this position on N-H group. Chlorine substituted sulfonamide III has a weaker antimicrobial activity than NO_2 _substituted ones due to less electron withdrawing ability of the chlorine. This is also supported by the results obtained by compound IV which has CH_3 _group in meta position. Methyl group donates electron to the benzene ring therefore reduces the ionization dramatically of the compound. The correlation of antimicrobial activity with chemical facts of the current study are also in line with biological activity results [19].

Antimicrobial activities of the sulfonamides depend on substituent and their position in the benzene ring. While electron releasing group decreases, electron withdrawing groups enhanced the activity of the sulfonamides against *S. aureus *isolates. Although sulfonamide-based therapy is generally effective, optimal treatment could be guided by antimicrobial susceptibility testing of isolates. Moreover, experimental data show that compound I may also be considered as a broad spectral effective sulfonamide at 128 μg (78% in total isolates) against MRSA and MSSA *S. aureus *isolates. Although 30 out of 50 *S. aureus *isolates showed resistance to oxacillin antibiotic, 21 of them were susceptible mainly to compound I and II.

## Conclusion

Sulfonamides are still an alternative option in order to cure MRSA staphylococci infections. However, increasing resistance against sulfonamides is a serious problem recently that has been taken attention. Therefore, there is need to have new chemicals for treatment of staphylococci and other bacterial infections. Outcome of the study has two noteworthy features. This study may help to suggest an alternative possible leading compound for development of new antimicrobial agents against MRSA and MSSA resistant *S. aureus*. It was also shown here that that clinical isolates of 50 *S. aureus *have various resistance patterns against to four sulfonamide derivatives. It may also be emphasized here that in vitro antimicrobial susceptibility testing results for *S. aureus *species need standardization with further studies and it should also have a correlation with in vivo therapeutic response experiments

## Abbreviations

MRSA: methicillin resistant *S. aureus*; MSSA: methicillin susceptible *S. aureus*; SXT: trimetoprim sulphamethoxazole; DMSO: dimethylsulfoxid

## Competing interests

The authors declare that they have no competing interests.

## Authors' contributions

All authors have read and approved the final manuscript.  RÖ participated in the analysis of the data and coordinated and drafted the manuscript.  YG carried out the antimicrobial activity tests and the data analysis.   YB participate the production of sulfonamide derivatives and approving the final manuscript.
